# Novel Feature for Catalytic Protein Residues Reflecting Interactions with Other Residues

**DOI:** 10.1371/journal.pone.0016932

**Published:** 2011-03-29

**Authors:** Yizhou Li, Gongbing Li, Zhining Wen, Hui Yin, Mei Hu, Jiamin Xiao, Menglong Li

**Affiliations:** College of Chemistry and State Key Laboratory of Biotherapy, Sichuan University, Chengdu, Peoples Republic of China; University of South Florida College of Medicine, United States of America

## Abstract

Owing to their potential for systematic analysis, complex networks have been widely used in proteomics. Representing a protein structure as a topology network provides novel insight into understanding protein folding mechanisms, stability and function. Here, we develop a new feature to reveal correlations between residues using a protein structure network. In an original attempt to quantify the effects of several key residues on catalytic residues, a power function was used to model interactions between residues. The results indicate that focusing on a few residues is a feasible approach to identifying catalytic residues. The spatial environment surrounding a catalytic residue was analyzed in a layered manner. We present evidence that correlation between residues is related to their distance apart most environmental parameters of the outer layer make a smaller contribution to prediction and ii catalytic residues tend to be located near key positions in enzyme folds. Feature analysis revealed satisfactory performance for our features, which were combined with several conventional features in a prediction model for catalytic residues using a comprehensive data set from the Catalytic Site Atlas. Values of 88.6 for sensitivity and 88.4 for specificity were obtained by 10fold crossvalidation. These results suggest that these features reveal the mutual dependence of residues and are promising for further study of structurefunction relationship.

## Introduction

Enzymes participate in various cellular processes by temporarily binding to reactants, significantly decreasing the activation energy required and accelerating the reaction. Enzyme structure provides an insight into such catalytic mechanisms. Since the advent of structure genomics projects, many enzyme structures have been explored however, determining the correlation of functional information with structural data and extrapolation to a catalytic mechanism remains a challenging task. Commonly, only a few amino acids in the active site of an enzyme are involved directly in such bioreactions. The prediction of catalytic residues in newly solved protein structures is highly desirable in structural proteomics and should help to further our understanding of catalytic mechanisms, which will be useful in protein engineering and in functional annotation.

Many studies have been devoted to the identification of active enzyme residues. Various features have been mined for active site description and can be roughly divided into several categories. Sequence [1] or structure [2] conservation analysis performs well in correlating residues with function because functionally important residues under high selective pressure usually exhibit a higher degree of conservation than other residues. Other properties for singling out active site residues have been investigated extensively. As reported by Bartlett [3], catalytic residues have relatively low solvent accessibility, tend to be charged or polar, are less flexible, are located in an appropriate cavity [4] and occur in coil regions. Moreover, most catalytic residues are involved in hydrogen bonding via amino acid main chains or side chains [3]. BenShimon *et al.* found that catalytic residues are frequently located close to the enzyme center [5]. Thus, sequential and structural features characterizing catalytic residues, such as residue type, physicochemical properties, hydrogen bonding, secondary structure, solvent accessibility and Bfactors, have been investigated in depth. Combination of these properties with information on evolutionary conservation has led to the development of numerous prediction models [6]–[14].

The threedimensional structural patterns of catalytic residues are usually shared by functionally similar enzymes and prediction can be made by searching for spatial patterns or templates resembling known catalytic sites [15]–[18]. Phylogenetic motifs, which are regions around key functional sites that are conserved in the overall phylogeny of a family, are promising for functional site prediction [19]. A mechanical study revealed high force constants for catalytic residues [20] and theoretical titration has proved useful by indicating the location of active sites [21]–[23]. Therefore, it is desirable to develop effective methods for describing such mutual restraints between catalytic and other residues, as well as the spatial environment around a catalytic residue.

Protein structure, as a type of complex system, can be analyzed by complex network approaches whereby the structure is represented as a residue contact network in which vertices are the residues and edges are their interactions. This method provides a novel insight into protein folding mechanisms, stability and function. Studies by Bagler *et al.* have indicated the smallworld and even scalefree [24] properties of such a network, which is independent of the structural class [25]. Vendruscolo *et al.* determined that a limited set of vertices with large connectivity, which they termed hubs, play a key role in protein folding [26]–[28]. In another study, hubs were defined as residues with more than four links that bring together different secondary structural elements, suggesting that these hubs contribute to both protein folding and stability [29]. Together, these studies have demonstrated that complex networks provide a convenient approach for systematic analysis of protein structure. Particularly high residue *closeness* values are associated with sequence conservation and reflect the key role in protein structure [30]. By definition, *closeness* score of a vertex is relative to its distances from all other vertices in a network, which reflects the global role of a residue in the global structure. These concepts are widely accepted as important features and have been combined with other features for the prediction of active sites [79], . In this study, several other network topological parameters were calculated and used to predict catalytic residues.

We determined the extent to which catalytic and noncatalytic residues differ in terms of their interactions with other residues. For this purpose, we developed the novel descriptor *description of network signal communication* *DNSC* for catalytic residues to reveal the effects imposed on catalytic residues by other residues. Here, effects from only a few key residues are taken into account, because proteins have evolved to a relatively optimized design that is robust to mutations and changes of the environment and extremely sensitive to perturbations at crucial sites. Moreover, Amitai *et al.* [30] and del Sol *et al.* [31] revealed that several central residues are vital for signal communication in the protein structure networks assumed for integration and transmittance of signals from and to the other residues. Our analysis demonstrates that these few residues are informative for the identification of catalytic residues.

To investigate the environmental influence on catalytic residues, a multilayer strategy based on the shortest path concept was used to characterize the environment surrounding catalytic residues. Several studies have revealed that catalytic residues are usually found in an unfavorable environment. Mutations of functional residues usually decrease enzyme activity but often increase stability at the same time [32], [33]. Thus, the free energy difference between naturally occurring and mutated amino acids at each position is useful for imposing constraints on functionally and structurally important residues [34]. We found that catalytic residues are affected by the outer layer the second and third layers environment and the effects of environmental features are steadily decreased as the layer number is increased.

Finally, a prediction model was constructed by combining these new features with several features reported earlier. Our model yielded satisfactory performance and was robust when implemented for a comprehensive noncatalytic residue set.

## Results

We used 10fold crossvalidation for the construction and testing of the model and the dataset was split at the protein level. To avoid an imbalance between catalytic and noncatalytic residues, the model was trained on a dataset with a ratio of 11 between catalytic and noncatalytic residues. Each residue was represented by a 130dimensional vector. Details of the features used for encoding a catalytic residue are given in Materials and Methods. The LIBSVM package was used for training the model http://www.csie.ntu.edu.tw/~cjlin/libsvm/ and we measured the results in terms of sensitivity recall, specificity, accuracy, precision and area under the curve AUC of the receiver operating characteristic ROC.

### Analysis of residue interactions for catalytic and noncatalytic residues

**Figure 1:**
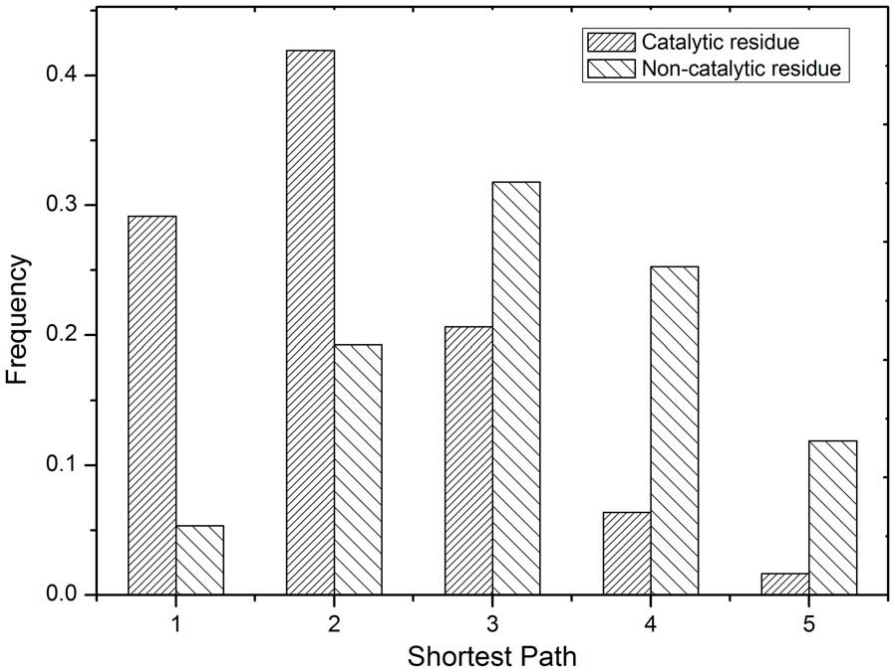
Observed frequency distribution of the shortest path between *keyAA*s and catalytic and non-catalytic residues.

First, we analyzed the interactions between *keyAAs* central amino acids in a protein structure network see Materials and Methods and catalytic and noncatalytic residues. The five highestranked *keyAA*s were investigated for each enzyme. Interactions with a distance of 5 were considered, whereas those 5 were regarded as uninformative and were not used. The suitability of this approach was confirmed by analysis. Fig. 1 shows that catalytic residues exhibited a strong tendency to approach *keyAA*s, especially with direct contact or is the *keyAA* itself or at an interval of one residue. The rates for these two cases were significantly lower for noncatalytic residues, at only 15 and 12 of the rates for catalytic residues, respectively. However, the opposite was true when the interaction distance increased. It was found that the length of the shortest path between noncatalytic residues and *keyAA*s was usually 2. These results are in accord with our hypothesis that *keyAA*s are vital for catalytic activity and their effect on catalytic residues decreases as the interaction distance increases. Each *keyAA* was the subject of detailed investigation in Fig S1. Interestingly, the difference of distribution for each *keyAA* was quite small suggesting that several residues play key roles during protein folding and more than one position participates in formation of the exquisite scaffold for effective activity, some of which have a direct and others an indirect effect.

**Figure 2:**
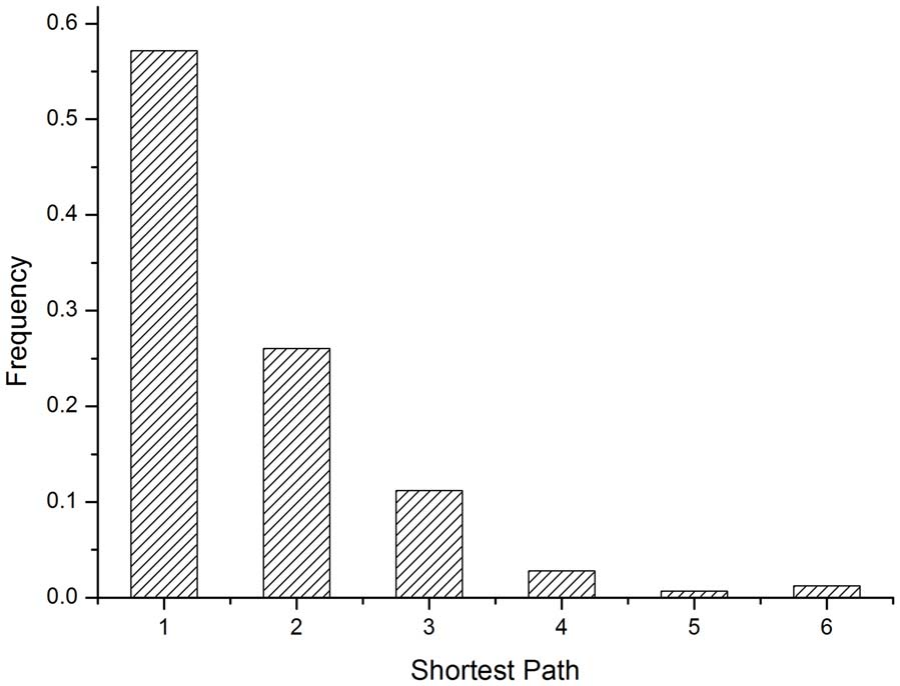
Observed frequency distribution of the shortest path between catalytic residues.

The shortest path between catalytic residues was analyzed Fig. 2. In most cases, intimate interactions were observed between catalytic residues. The fraction of interactions with direct contact and those with an interval of one residue are 57 and 26, respectively, which indicates collaboration between catalytic residues for effective function. In this method, some catalytic residues were also scored highly by *closeness* and were therefore treated as *keyAA*s. In this sense, correlations among catalytic residues are also, at least partially, implied by *DNSC*.

**Figure 3:**
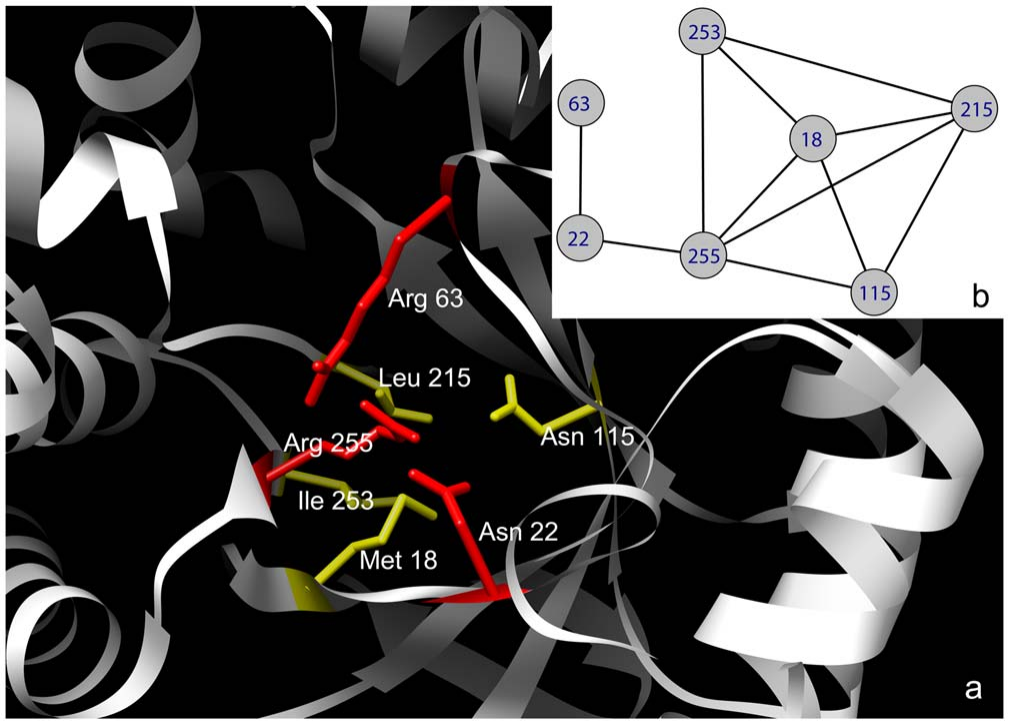
The spatial structure and local contact network for *dihydropteroate synthase* (1aj0). (a) The local structure of the catalytic residues (yellow) and *keyAA*s (red). (b) The local contact network for the catalytic residues and *keyAA*s. Here, Asn22, Arg63, and Arg255 are catalytic residues, which were observed adjacent to *keyAA*s Met18, Asn115, Leu215, Ile253 and Arg255 and their interactions are shown.

A detailed case study of *dihydropteroate synthase* PDB1aj0 is presented Fig. 3. Residues Met18, Asn115, Leu215, Ile253 and Arg255 are distant in the sequence but spatially close and were identified as the *keyAAs* in this structure. The catalytic site consists of the catalytic residues Asn22, Arg63 and Arg255, which was observed adjacent to *keyAA*s. The local interaction network for *keyAA*s and catalytic residues is shown in Fig. 3b. Arg255 was determined as a *keyAA* with direct interactions with other *keyAA*s. Asn22 has direct contact with Arg255, whereas the length of its shortest path to the other *keyAA*s is 2. Arg63 was far from the *keyAA*s however, close connections were found between this and the two other catalytic residues.

### Feature evaluation

To gauge the resolution limits of classification by our novel features in this prediction task, each feature alone was used to construct a prediction model for catalytic residues and compared to other features used in earlier studies Table 1. Models based on these individual features were trained using the scheme described above. *DNSC* achieved an average sensitivity of 69.6 and specificity of 79.0. Its specificity is 6 higher than the value for *closeness*. This means that, for identification of a catalytic residue, these limited *keyAA*s are as informative as all the rest of the residues in a protein together suggesting that not all residues within a protein are equally important for structure andor function. The *conservation score* performed best, with 76.4 sensitivity and 82.5 specificity. Catalytic residues are usually provided by charged and polar residues. So, the *AAIdentity* a 20dimensional vector used to denote a residue type performed well in identifying catalytic residues. However, it determined only 67.4 of noncatalytic residues.

**Table 1:** Performance for each feature by 10-fold cross-validation.

Feature set	Sensitivity	Specificity	Accuracy	AUC
*Conservation*	76.4	82.5	82.5	0.829
*Layer1* a	82.8	80.2	80.2	0.894
*Layer2* b	70.3	70.3	70.3	0.778
*Layer3* c	68.4	69.0	69.0	0.749
*Neigs* d	84.0	83.9	83.9	0.907
*AA Identity*	75.8	67.4	67.5	0.753
*Network parameter*	77.1	76.7	76.7	0.835
*Closeness*	76.7	73.2	73.2	0.826
*DNSC*	69.6	79.0	79.0	0.781

a Environmental features in the first layer.

b Environmental features in the second layer.

c Environmental features in the third layer.

d Environmental features of all layers.

In Table 1, *Layer*1, *Layer*2 and *Layer*3 denote environmental features in layers 1, 2 and 3, respectively. *Layer*1 achieved the best performance with 82.8 sensitivity and 80.2 specificity. The performance of environmental features decreased steadily as the layer number increased. The environmental features of *Layer2* correctly predicted 70.3 of catalytic residues and 70.3 of noncatalytic residues. This is in agreement with earlier reports and highlights the different selection pressure on the spatial environment of catalytic residues to maintain an efficient scaffold. The performance was enhanced when using features of all three layers, with 84.0 sensitivity and 83.9 specificity, which imply the dependence of catalytic residues on the neighboring environment. Network topological features were found to predict 76 of residues correctly. The *AUC* values for these features were calculated and are given in Table 1. These results suggest the feasibility of studying structurefunction relationship by revealing interactions between several residues.

### CrossValidation and Feature Selection

**Figure 4:**
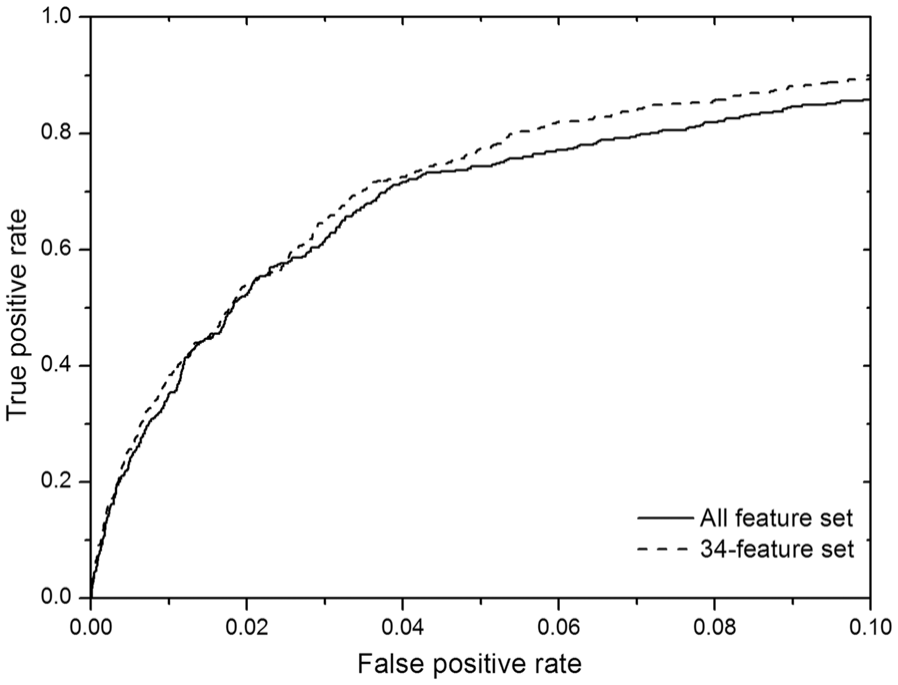
The ROC curves for the all-feature set and the 34-feature set.

A prediction model was constructed for catalytic residues, by a combination of *DNSC* and network topological and environmental features with several conventional features for a detailed description see Materials and Methods. The average results over the 10fold crossvalidation are given in Table 2 and the sensitivity and specificity were 88.6 and 88.4, respectively. The ROC performance is shown in Fig. 4. Using our data set, our method achieved a recall value of 66.8 at a precision of 15.

**Table 2:** Performance for each feature set by 10-fold cross-validation.

Feature set	Sensitivity	Specificity	Accuracy	AUC
All	88.6	88.4	88.4	0.945
88	89.5	88.7	88.7	0.951
70	91.3	88.3	88.3	0.952
34	91.1	88.8	88.8	0.954

Here, the first 34, 70 and 88 features yielding the greatest contributions were selected to construct the prediction model for catalytic residues.

To further analyze the impact of features on prediction performance and choose an optimized subset, feature evaluation was done by using the select attributes module in Weka 3.6.1 [35] according to the square of the weight assigned by the SVM [36]. In this step, elements in the vector of *DNSC* and *AAIdentity* features were treated individually. The merit of features is given in Table S1. It is evident that *conservation score*, *polar* and *closeness* make the greatest contributions to prediction. The environmental features, especially those in the first and second layers, appear to be very important for catalytic residues. Network topological features and physiochemical properties of amino acids in the first layer made great contributions to prediction. High scores were observed also for *AAIdentity*, *PSSM* and *weighted frequencies*. Interestingly, *accessible surface area* and *relative accessible surface area* made limited contributions to prediction, although they were used as the major predictors in earlier studies [30], [37].

The first 34, 70 and 88 features yielding the greatest contributions were selected to develop prediction models for catalytic residues. A 10fold crossvalidation was done and the average results are given in Table 2. Sensitivity and *AUC* were improved when the uninformative features were eliminated and both sensitivity and specificity were increased by using the 88feature set,. The sensitivity value of the 70feature set and the 34feature set was enhanced significantly by 3 however, specificity for the 70feature set was slightly decreased. Using the 34feature set, the best performance was obtained with a sensitivity value of 91.1 and a specificity value of 88.8. In this set, features in the second and third layer environment as well as the element of *DNSC* were included. The corresponding *AUC* values are 0.945, 0.951, 0.952 and 0.954. The *ROC* performance is shown in Fig. 4 where the curve for the allfeature set can be seen to be dominated by that for the 34feature set.

### Model Evaluation

Six benchmark datasets that allow direct comparison with wellestablished methods were used to assess the performance of our method in Table 3. Models constructed by using the allfeature set *model1* and the 34feature set *model2* were used for comparison. We used 10fold crossvalidation on these datasets except for the data set from *Chea et al.* [37], on which 5fold crossvalidation was used instead. For the three datasets from *Youn et al.* [12], significant improvement of recall was observed for our methods at a precision corresponding to that reported by *Youn et al.* Our methods attained 10 greater recall at a precision of 14.9, *Chea et al.* obtained a recall value of 54.0, while *model1* and *model2* found 67.2 and 66.4 recall, respectively. *Gutteridge et al.* achieved a recall of 56.0 at a precision of 14.0 and the performance was enhanced remarkably by using spatial clustering with a recall of 68.0 and a precision of 16.0. We found our methods also performed well on their data set with 10 greater recall at 14.0 precision. The *model2* achieved a recall even slightly higher than the refined result reported by *Gutteridge et al. Petrova et al* [7] reported a high recall value of 90 at a precision of 7. For this dataset, the recall was 64.1 for *model1* and 67.3 for *model2* at a precision of 18.0 on the basis of the crossvalidation. The satisfactory performance confirmed the robustness of our method. Thus, it is reasonable to believe that identifying catalytic residues by analyzing their interactions with other residues is both feasible and promising.

**Table 3:** Comparison with competing methods.

MethodData set	EF family^a^	EF superfamily^b^	EF fold^c^	HA superfamily^d^	NN^e^		PC^f^
	gRecall^18.5^	Recall^16.9^	Recall^17.1^	Recall^14.9^	Recall^14.0^	Recall^16.0^	Recall^7.0^
Allfeature	60.62	63.94	63.3	67.2	69.8	64.2	64.1
34feature set	66.01	59.24	60.87	66.4	73.4	68.7	67.3
Competing methods	57.02	53.93	51.11	54.0	56.0	68.0	90.0

a Results on the data set from Youn *et al.* at the SCOP family level.

b Results on the data set from Youn *et al.* at the SCOP superfamily level.

c Results on the data set from Youn *et al.* at the SCOP fold level.

d Results on the data set from Chea *et al.* at the SCOP superfamily level.

e Results on the data set from Gutteridge *et al.*

f Results on the data set from Petrova *et al.* at the SCOP superfamily level.

g Recall at the corresponding precision reported in earlier studies.

## Discussion

Identification of catalytic residues can help to further our understanding of the catalytic mechanism of biological reactions. A great deal of effort has been devoted to the development of effective prediction models, for which good descriptors are a prerequisite. Complex networks enable systematic analysis of enzyme structure. On the basis of the results of the present study, we propose a novel feature, *DNSC*, which is based on an enzyme structure network. Unlike the reported *closeness* centrality, this feature focuses on the communication between *keyAA*s, instead of all the other residues and catalytic residues. Its satisfactory performance suggests its promise in describing the correlation between residues. Moreover, environmental parameters, especially those in *Layer1* and *Layer2*, do help to discriminate between catalytic and noncatalytic residues. The limited contribution from *Layer3* implies that more variation might occur in residues far from the catalytic site.

Our results confirm that systematic analysis has great potential for the analysis of protein structure. But the present study is only an initial step in this direction. Further studies will be complicated by virtual variations in protein structure. Residues interact mutually in various ways, including hydrogen bonding, interactions and hydrophobic interactions. The fact that two residues can be connected by more than one shortest path should be considered. Earlier research revealed that catalytic residues tend to be located in unfavorable environments which might be an important clue in distinguishing catalytic from neighboring residues. In conclusion, investigation of the correlations among residues and their links to protein structure and function remains an important challenge.

## Materials and Methods

### Dataset

The study data set was derived from PDB according to annotations in the Catalytic Site Atlas CSA database version 2.2.10 [38]. An enzyme entry was selected if i its PDB structure resolution is better than 2.5 and ii it was taken from the literature. The final data set consists of 140 enzyme structures that cover the six toplevel EC classifications and is filtered at the SCOP superfamily level. For comparison with previous methods, six benchmark data sets were prepared, including those from *Petrova et al.* [7] and Gutteridge *et al.* [6], the SCOP superfamily dataset from *Chea et al.* [37], and three datasets at different SCOP levels from *Youn et al.* [12].

### Protein Structure Network

In this study, each chain was considered as a selfgoverned complex system, regardless of the possible interactions between chains. An enzyme structure was modeled as a network system in which residues are the vertices and connections between residues are the edges. Here, edges are defined such that two residues have a connection if the distance between any pair of atoms, one from each residue, is smaller than the sum of their van der Waals radii plus a threshold value of 2.

### Feature extraction

#### Description of Network Signal Communication DNSC

**Figure 5:**
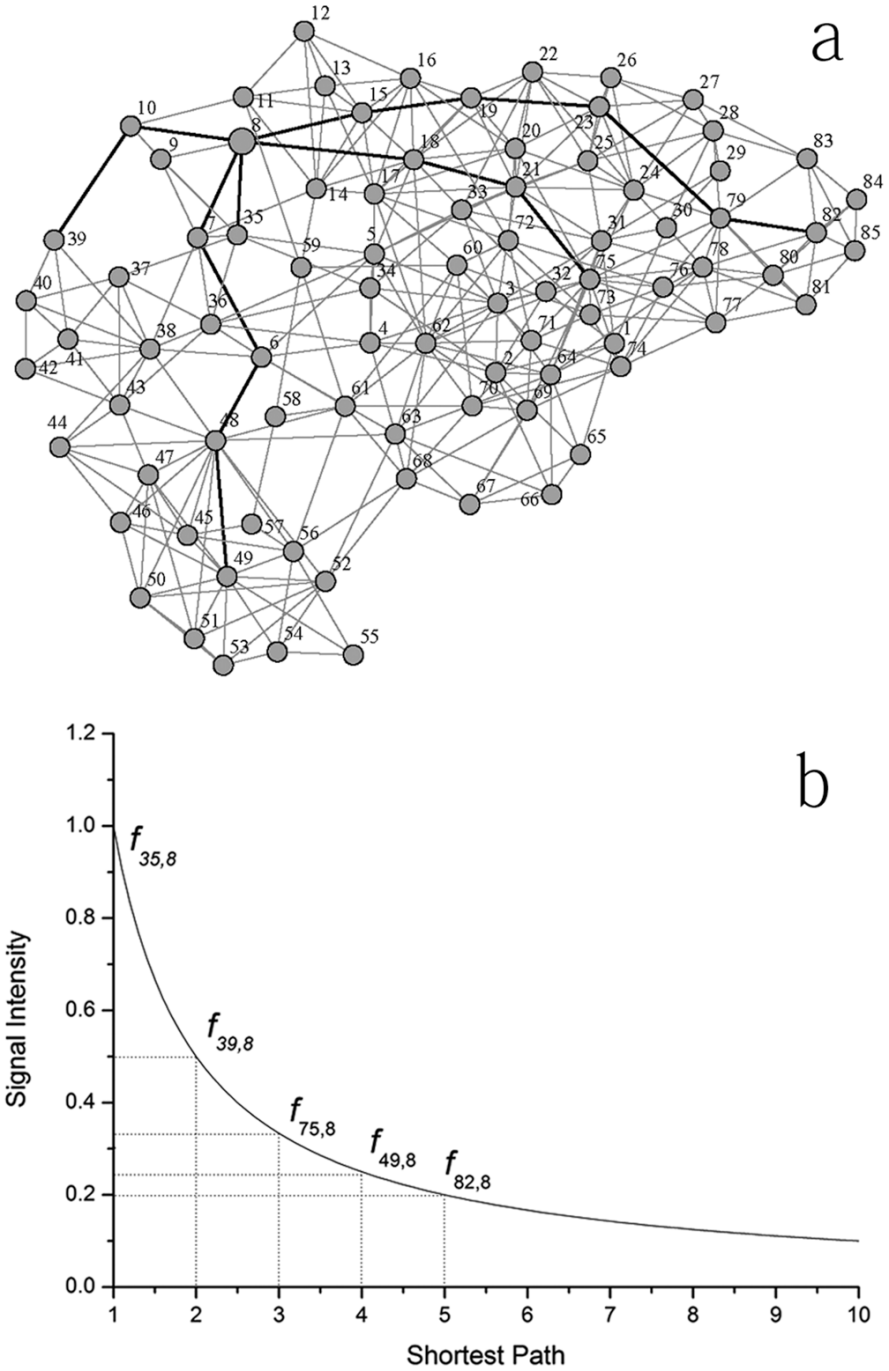
Description of residue interaction based on the protein structure network. (a) Network representation of Glutaredoxin 1 (1QFN). (b) Depiction of signal attenuation model by power function. Here, vertex 8 was taken for advance and vertices 35, 39, 49, 75 and 82 were selected with the different shortest path length to vertex 8.

The protein structure was treated as a selfgoverned complex system, and the active residue was treated as a terminus of the signal network i.e. the protein structure network that receives informative signals from other residues we call them signal sources in this context via direct andor indirect contacts. We attempted to quantify the intensity of these signals by postulating that the intensity decreases as distance between signal sources increases. It arises from the physiochemical intuition that a residue has stronger impacts on its closer neighbors. The signal transduction mode was generated for the protein structure network constructed according to the following assumptions i signal flows along the shortest path and ii the signal intensity is attenuated when passing through a vertex. Here, we postulate that the signal is regularly dampened and the intensity on reaching a vertex can be calculated as
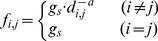
(1)where *f_i_*_,*j*_ is the signal intensity at vertex *j* received from vertex *i* represented as a function of *d_i_*_,*j*_, the shortest path length between *i* and *j*. A power function was used to simulate signal attenuation with the exponential of *a* here *a*1 *g_s_* is the signal intensity at the signal source that was assumed to be 1 in this study. To illustrate this attenuation, the network representation of the whole structure of *glutaredoxin 1* 1qfn is shown in Fig. 5a. The bold line depicts the five shortest paths to Arg8 and the signal intensity along these paths is shown in Fig. 5b.

Although most residues are coupled by a shortest path of either long or short distance in this context, distance refers to the length of the shortest path, only information from residues playing a major role in protein structure key residues was considered. As reported, residues with high *closeness* values are considered to play a key role in protein folding. So, we used *closeness* of a residue as the measure of its structural importance. The question remains of how large a threshold is appropriate It is hard to establish a rigorous criterion because of the variety of protein structures. In the present study, residues in a protein were ranked by *closeness* and the topranked residues were taken into account in this context we call them *keyAA*s. Five *keyAA*s were used for protein encoding and this yielded satisfactory performance. A residue can therefore be described by a vector of signal intensities. For example, the catalytic residue Lys34 in *DNA ligase* 1a0i can be represented by the vector
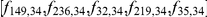
where residues Leu149, Trp236, Glu32, Leu219 and Tyr35 are the top residues ranked by *closeness*.

#### Conventional Properties of Residues

Several conventional features were used to characterize the residues, including *sequence conservation*, *amino acid type*, *polarity*, *hydrophobicity*, *volume*, *accessible surface area*, *relative accessible surface area*, *secondary structure*, *degree*, *cluster coefficient*, *hubscore*, *cocitation*, *coreness*, *constraint*, *betweenness* and *closeness*. The last eight parameters were derived from the protein structure network. A detailed description of the features is given below.

#### Sequence conservation

Residues essential for protein function are conserved during evolution. Thus, conservation scores were calculated as one of the most important properties. Positionspecific iterated BLAST PSIBLAST [39] has been generally used in studies on proteomics. Here, it was implemented against the 90 nonredundant protein database with an Evalue cutoff of 1E3 and 3 iterations. The output positionspecific scoring matrix PSSM and weighted observed percentage were used to characterize a catalytic residue. Furthermore, the conservation score is defined as
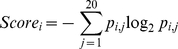
(2)where p*_i,j_* is the frequency of amino acid *j* at position *i*. A lower value suggests lower entropy more conserved at a position and vice versa.

#### Amino Acid Properties

As defined by Bartlett et al. [3], catalytic residues are directly involved in catalytic reactions as donors or acceptors or assist in reactions by exerting effects on the catalytic mechanism or the structural stability of the enzyme. Thus, residues occupying catalytic sites are usually polar or charged. A feature called *AAType* encodes charged DEKHR, *polarity* CNQSTY and hydrophobic residues AFGILMPVW as 0 0, 0 1 and 1 0, respectively. Physicochemical properties such as *polarity* and *hydrophobicity* and *volume* are used to further characterize catalytic residues quantitatively.

#### Accessible surface area and secondary structure

It is considered that catalytic residues are usually restricted in their correct position for enzyme function. Thus, in most cases catalytic residues exhibit a relatively low level of solvent accessibility. Accordingly, the *accessible surface area* and the *relative accessible surface area* were calculated for residues using DSSP [40]. As mentioned above, a single chain was regarded as an independent unit. Thus, values for chains were calculated separately, with ligands excluded for protein complexes. The secondary structure type for a residue was also derived by DSSP.

#### Network parameters

Translation of a protein structure to a network facilitates systematic analysis of the protein structure. In the present study, the igraph version 0.5.1 software package [41] was used to calculate network parameters. Eight network parameters, *degree*, *cluster coefficient*, *hubscore*, *cocitation*, *coreness*, *constraint*, *betweenness* and *closeness*, were used to describe residues. These parameters are described in detail by Watts and Newman *et al.* [42]–[45]

#### Layered Description of the Structural Environment

**Figure 6:**
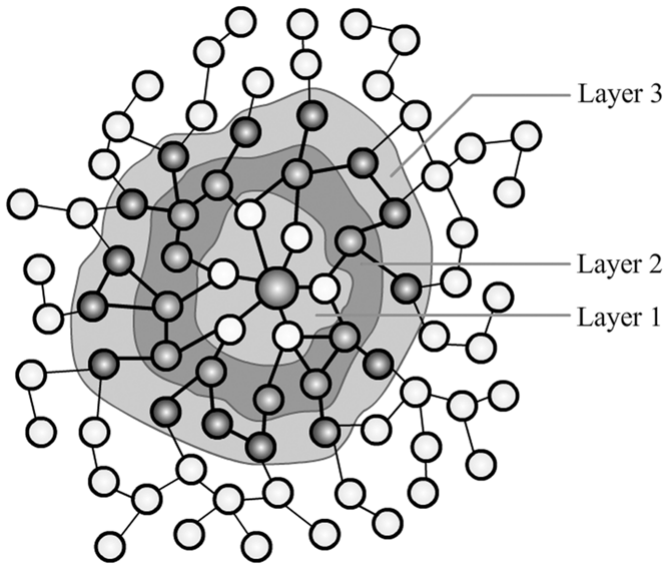
Layered description of the structural environment. Layer 1 consists of residues with a shortest path of 1; i.e. in direct contact with the catalytic residues. Layer 2 and 3 consist of residues with shortest paths of 2 and 3, respectively.

Functional residues tend to be located in unfavorable environments and therefore do not always satisfy structural requirements. Thus, it would be useful to introduce environmental parameters into schemes for the identification of catalytic residues. Moreover, as observed by Bartlett et al. [3], residue conservation is inversely proportional to the distance from catalytic residues. Thus, it is reasonable to believe that catalytic residues are more affected by residues that are closer. For this reason, we used a layered description of the structural environment. The structural network constructed in this study makes partition easy to implement. Based on the shortest path to catalytic residues, the surrounding residues naturally fall into three layers. The first layer consists of residues with a shortest path of 1 namely, in direct contact with the catalytic residues. The second and third layers comprise residues with shortest paths of 2 and 3, respectively. A sketch map of this layered description is shown in Fig. 6.

The average values for all these features were used to reflect the physicochemical properties of surrounding residues and their importance in maintaining protein structure. Thus, a single layer of the environment can be represented simply by a 14dimensional vector. For each, a suffix of the layer number is added to each feature name as a distinctive mark. Thus, the layered environment was encoded by a 42dimensional vector.

## Supporting Information

Figure S1 **Observed frequency distribution of the shortest path between** ***keyAA*** **and catalytic and noncatalytic residues.** Distribution of a shortest path to the first ranked *keyAA* b shortest path to the second ranked *keyAA* c shortest path to the third ranked *keyAA* d shortest path to the fourth ranked *keyAA* e shortest path to the fifth ranked *keyAA*.
TIF

Table S1 **The merit score for each feature.**
DOC
