# Supplementary figures and images for: Novel Feature for Catalytic Protein Residues Reflecting Interactions with Other Residues
Source: PLoS One. 2011 Mar 29;6(3):e16932. doi: 10.1371/journal.pone.0016932 (PMC3066176; doi:10.1371/journal.pone.0016932)

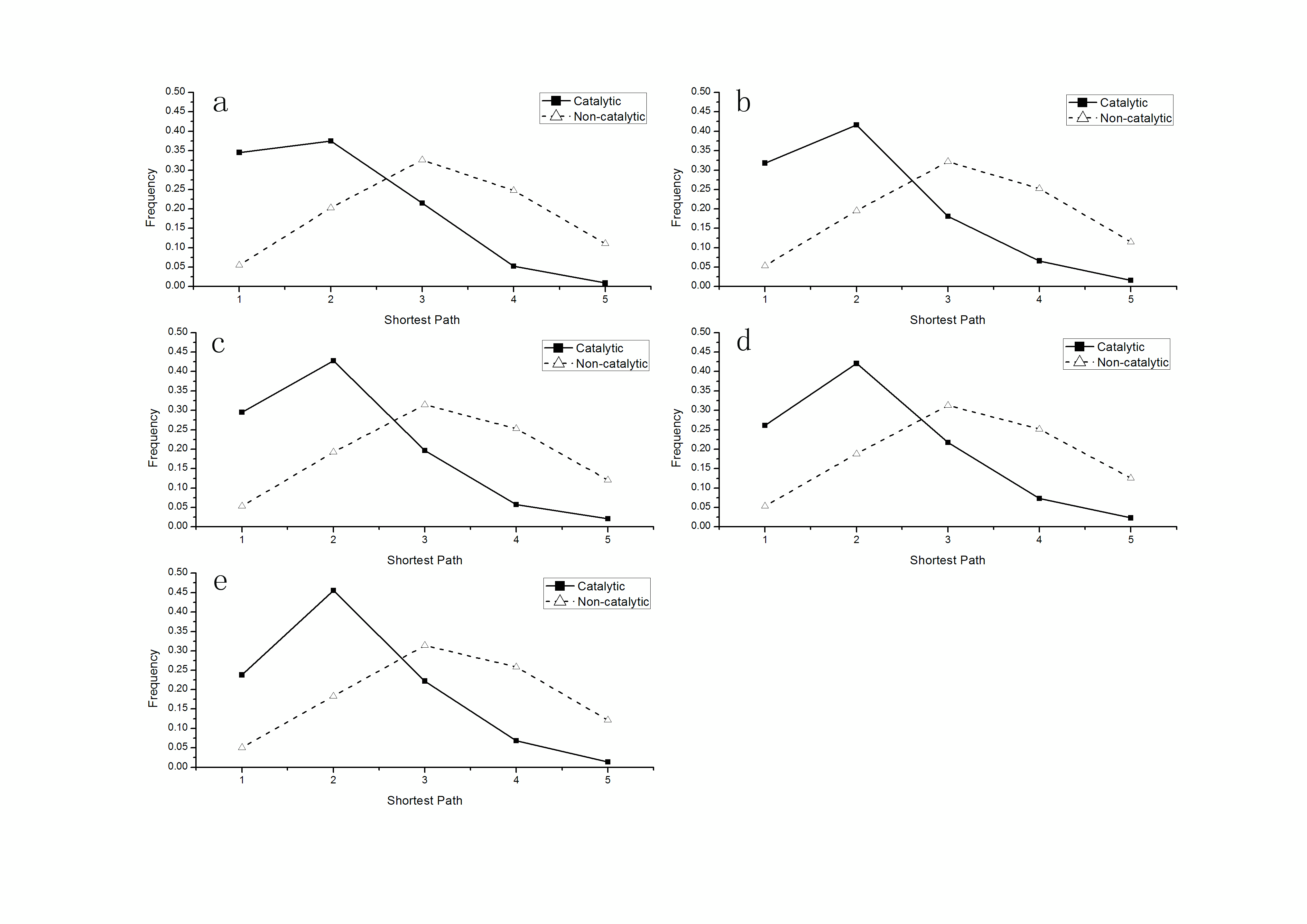

Supplement: Figure S1 — Observed frequency distribution of the shortest path between keyAA and catalytic and noncatalytic residues. Distribution of a shortest path to the first ranked keyAA b shortest path to the second ranked keyAA c shortest path to the third ranked keyAA d shortest path to the fourth ranked keyAA e shortest path to the fifth ranked keyAA. TIF [file pone.0016932.s001.tif]
