# Supplementary material for: Novel Feature for Catalytic Protein Residues Reflecting Interactions with Other Residues
Source: PLoS One. 2011 Mar 29;6(3):e16932. doi: 10.1371/journal.pone.0016932 (PMC3066176; doi:10.1371/journal.pone.0016932)
Supplement: Table S1 — The merit score for each feature. DOC [file pone.0016932.s002.doc]

Table S1 The merit score for each feature

| **Merit** | **Feature** |
| --- | --- |
| **130** | Conservation Score |
| **128.6** | Polar |
| **127.6** | Closeness |
| **125.5** | PSSM(1) |
| **124.5** | AA_Identity(7) |
| **124** | AA_Identity(2) |
| **121.3** | Frequency(15) |
| **119.6** | Frequency(5) |
| **119** | Coreness_L1 |
| **118.3** | Volume_L2 |
| **114.5** | PSSM(18) |
| **111.2** | Polar_L1 |
| **110.8** | Constraint_L1 |
| **110.7** | Hydrophobicity |
| **109.8** | Frequency(1) |
| **109.7** | Volume |
| **109.5** | Secondary Structure(5) |
| **109** | Conservation Score_L1 |
| **108.8** | Cocitation_L1 |
| **106.8** | PSSM(16) |
| **106.8** | Hydrophobicity_L1 |
| **105.8** | AA_Type(1) |
| **105.4** | Volume_L1 |
| **104.6** | Hydrophobicity_L2 |
| **103.5** | Polar_L2 |
| **102** | Frequency(16) |
| **100.3** | Secondary Structure(2) |
| **99.9** | Betweenness_L1 |
| **98.9** | Degree_L1 |
| **97.9** | PSSM(15) |
| **96.9** | Frequency(17) |
| **93.9** | Conservation Score_L3 |
| **92** | DNSC(5) |
| **90.4** | Betweenness_L3 |
| **89.8** | Betweenness |
| **88.2** | AA_Identity(5) |
| **86.5** | Frequency(9) |
| **84.7** | Hubscore_L2 |
| **84.6** | AA_Identity(4) |
| **81.8** | PSSM(7) |
| **81.6** | PSSM(8) |
| **80.5** | PSSM(17) |
| **80.3** | Volume_L3 |
| **80.1** | Closeness_L1 |
| **76.3** | PSSM(3) |
| **74.3** | Frequency(12) |
| **74.2** | Closeness_L3 |
| **74.2** | Frequency(3) |
| **74** | Relative Accecible Surface Area_L3 |
| **73.2** | Accecible Surface Area_L1 |
| **72.8** | Clustering Coefficients |
| **72.4** | PSSM(4) |
| **71.7** | Conservation Score_L2 |
| **71.5** | Clustering Coefficients_L1 |
| **70.6** | Coreness_L2 |
| **70.1** | Frequency(7) |
| **68** | Frequency(19) |
| **67** | Clustering Coefficients_L2 |
| **66.3** | AA_Identity(15) |
| **65.7** | PSSM(12) |
| **64.9** | Coreness |
| **63.1** | Clustering Coefficients_L3 |
| **61.5** | Frequency(18) |
| **61** | Polar_L3 |
| **59.7** | AA_Identity(13) |
| **59.6** | Relative Accecible Surface Area |
| **58.5** | Hydrophobicity_L3 |
| **58.4** | Degree_L3 |
| **58.3** | Hubscore_L3 |
| **57.3** | Frequency(13) |
| **54.7** | PSSM(19) |
| **53.6** | PSSM(13) |
| **53.6** | Coreness_L3 |
| **53** | AA_Identity(17) |
| **52.5** | Relative Accecible Surface Area |
| **51.9** | DNSC(4) |
| **49.3** | Secondary Structure(3) |
| **48.7** | Frequency(4) |
| **48.3** | AA_Identity(20) |
| **46.5** | Frequency(8) |
| **46.4** | Frequency(6) |
| **46.3** | Frequency(11) |
| **45.8** | Frequency(20) |
| **45.5** | AA_Identity(6) |
| **45.3** | AA_Identity(12) |
| **45** | DNSC(2) |
| **45** | Accecible Surface Area_L3 |
| **44.4** | Degree |
| **44.2** | PSSM(20) |
| **44.2** | Hubscore_L1 |
| **43.8** | Betweenness_L2 |
| **43.8** | PSSM(2) |
| **43.7** | Secondary Structure(7) |
| **43.5** | Constraint |
| **43.2** | Frequency(14) |
| **41.3** | DNSC(1) |
| **40.2** | Frequency(2) |
| **39.7** | AA_Identity(16) |
| **39.1** | PSSM(11) |
| **39.1** | Frequency(10) |
| **38.9** | Closeness_L2 |
| **37.6** | AA_Identity(9) |
| **37.5** | Accecible Surface Area_L2 |
| **36.1** | PSSM(9) |
| **35** | Secondary Structure(4) |
| **34.8** | AA_Identity(19) |
| **34.1** | PSSM(6) |
| **34** | AA_Type(2) |
| **32.5** | AA_Identity(11) |
| **31.3** | AA_Identity(3) |
| **31.2** | AA_Identity(10) |
| **30.1** | Degree_L2 |
| **29.8** | PSSM(5) |
| **29.1** | AA_Identity(8) |
| **26.6** | Relative Accecible Surface Area_L1 |
| **26.1** | DNSC(3) |
| **25.9** | AA_Identity(1) |
| **25.8** | Cocitation_L3 |
| **25.6** | Constraint_L2 |
| **25.2** | PSSM(14) |
| **21.1** | Relative Accecible Surface Area_L2 |
| **20.7** | Cocitation |
| **20.6** | Cocitation_L2 |
| **20** | Constraint_L3 |
| **19.5** | AA_Identity(18) |
| **17.9** | Accecible Surface Area |
| **17** | Secondary Structure(6) |
| **16.7** | PSSM(10) |
| **16.4** | AA_Identity(14) |
| **16.1** | Hubscore |
| **130** | Conservation Score |

The merit scores were calculated by using the attribute selection module in Weka 3.6.1. The suffix L1, L2 and L3 denote the environment of the first layer, second layer and third layer, respectively. The number in the parentheses indicates the element order in the corresponding feature vector.
